# T cell-related ubiquitination genes as prognostic indicators in hepatocellular carcinoma

**DOI:** 10.3389/fimmu.2024.1424752

**Published:** 2024-06-11

**Authors:** Chaobo Chen, Zheng Chen, Zheyu Zhou, Hui Ye, Shaohui Xiong, Weidong Hu, Zipeng Xu, Chen Ge, Chunlong Zhao, Decai Yu, Jiapei Shen

**Affiliations:** ^1^ Department of General Surgery, Xishan People’s Hospital of Wuxi City, Wuxi, China; ^2^ Department of Hepatobiliary and Liver Transplantation Surgery, Drum Tower Hospital Affiliated to Nanjing University School of Medicine, Nanjing, China; ^3^ Department of General Surgery, Nanjing Drum Tower Hospital, Chinese Academy of Medical Sciences & Peking Union Medical College, Graduate School of Peking Union Medical College, Nanjing, China; ^4^ Department of Anesthesiology, ZhongDa Hospital, Southeast University, Nanjing, Jiangsu, China; ^5^ Department of Cardiology, Huzhou Central Hospital, Huzhou, China; ^6^ Department of Infectious Diseases, Huzhou Central Hospital, Huzhou, China

**Keywords:** HCC, ubiquitination modification, T cell, immunotherapy response, prognosis, UBE2E1

## Abstract

**Background:**

T lymphocytes, integral to the adaptive immune system, wield pivotal influence in bolstering anti-tumor responses, and are strictly regulated by ubiquitination modification. The objective of this investigation was to devise a novel prognostic and immunotherapeutic efficacy predictor for hepatocellular carcinoma patients utilizing T cell-related ubiquitination genes (TCRUG).

**Method:**

The single-cell RNA sequencing (scRNA-seq) data and bulk RNA data of HCC patients are derived from the GEO database and TCGA database. Based on the processing of scRNA-seq, T cell marker genes are obtained and TCRUG is obtained. Further combined with WGCNA, differential analysis, univariate Cox regression analysis, LASSO analysis, and multivariate Cox regression analysis to filter and screen TCRUG. Finally construct a riskscore for predicting the prognosis of HCC patients, the predictive effect of which is validated in the GEO dataset. In addition, we also studied the correlation between riskscore and immunotherapy efficacy. Finally, the oncogenic role of UBE2E1 in HCC was explored through various *in vitro* experiments.

**Result:**

Based on patients’ scRNA-seq data, we finally obtained 3050 T cell marker genes. Combined with bulk RNA data and clinical data from the TCGA database, we constructed a riskscore that accurately predicts the prognosis of HCC patients. This riskscore is an independent prognostic factor for HCC and is used to construct a convenient column chart. In addition, we found that the high-risk group is more suitable for immunotherapy. Finally, the proliferation, migration, and invasion abilities of HCC cells significantly decreased after UBE2E1 expression reduction.

**Conclusion:**

This study developed a riskscore based on TCRUG that can accurately and stably predict the prognosis of HCC patients. This riskscore is also effective in predicting the immune therapy response of HCC patients.

## 1 Introduction

Primary liver cancer claims approximately 830,000 lives annually, making it one of the deadliest cancers (1). Hepatocellular carcinoma (HCC) constitutes around 90% of primary liver cancer cases (2, 3). It is noteworthy that the incidence and mortality rates of liver cancer are projected to continue increasing (4). As most HCC patients are diagnosed at advanced stages, curative treatments such as surgery are no longer feasible. Immunotherapy has emerged as a promising novel approach for treating advanced-stage HCC patients, although it remains highly challenging (5, 6). Despite this, the prognosis for HCC patients remains dismal, underscoring the need to focus on the immune microenvironment to identify robust biomarkers for predicting prognosis and response to immunotherapy in HCC patients.

The tumor microenvironment (TME) of HCC is a complex ecosystem involving various cell types, including tumor cells, immune cells, and fibroblasts (7). The interactions between these cells play a crucial role in tumor progression (8). In particular, dysfunction of the innate and adaptive immune systems may lead to the generation of an immunosuppressive TME, resulting in immune evasion (9, 10). T cells, as key members of the adaptive immune response, play an important role in HCC. However, continuous stimulation of tumor antigens often leads to T cell dysfunction, affecting T cell-mediated antitumor immune response (11). Some scholars believe that the gene regulatory programs controlling T cell dysfunction are highly conserved, suggesting that while the specific mechanisms of T cell dysfunction may vary among different tumor types, they may share common regulatory mechanisms at the genetic level (12). Given the critical role of T cells in HCC and their key role in immune evasion, it is important to study comprehensively the combined effects of T cell marker genes in HCC.

Activation of the adaptive immune system can affect various physiological processes, including protein homeostasis, antigen processing, signal transduction, etc., all of which are regulated by ubiquitination modification (13). Ubiquitination modification is an important post-translational modification, which plays an indispensable role in ensuring the normal function of immune cells (14). Studies have shown that silencing the MYH9 gene can inhibit the ubiquitination and degradation process of GSK3β induced by HBx, thereby achieving the effect of inhibiting the tumor stemness of hepatocellular carcinoma (15). Additionally, by promoting the ubiquitination of UVRAG, SMURF1 enhances the function of autophagosomes, effectively inhibiting the growth of hepatocellular carcinoma (16). Therefore, in this study, we focus on exploring the ubiquitination family molecules in T cell marker genes. We believe that analyzing T cell function from the perspective of ubiquitination is a highly promising research direction.

In this study, we performed dimensionality reduction on scRNA-seq data to reveal unique immune cell subtypes in HCC and identified 3050 T cell marker genes. In addition, we further integrated bulk RNA data to construct a riskscore that accurately predicts the prognosis and immunotherapy outcomes of HCC patients. Most importantly, we identified the key gene UBE2E1’s oncogenic role in HCC *in vitro*.

## 2 Methods and materials

### 2.1 Dataset download

Initially, transcriptome data and clinical information of liver cancer were screened from the TCGA database (https://portal.gdc.cancer.gov/), obtaining a total of 374 liver cancer samples and 50 normal samples. The clinical information included data on survival time, survival status, age, sex, and TNM staging. Additionally, liver cancer bulk datasets GSE14520 and GSE36376, as well as scRNA-seq dataset GSE149614, were downloaded from the GEO database (https://www.ncbi.nlm.nih.gov/). After strict screening, a total of 221 liver cancer samples were selected from the GSE14520 dataset for further analysis, while the GSE149614 dataset contained 10 liver cancer samples.

### 2.2 Single cell sequencing analysis

Single-cell sequencing analysis is an advanced high-throughput sequencing technology aimed at deeply sequencing the transcriptomes of individual cells. Through this technology, we can gain insights into the functional differences and expression characteristics among different cell types, as well as the heterogeneity within individual cells. At the beginning of the analysis, we conducted meticulous screening of the sequencing data to ensure data quality and excluded poorly performing samples. Subsequently, the Seurat package was used to further analyze the filtered dataset (17), and cell samples were reasonably clustered using PCA and t-SNE dimensionality reduction techniques. To further resolve cell types, the SingleR package was employed for precise cell type annotation and key gene selection in single-cell data (18).

### 2.3 WGCNA analysis

WGCNA is a method that assists researchers in extracting key information from high-throughput gene expression data to reveal the structure and function of gene regulatory networks (19). The functions of WGCNA include identifying gene modules, identifying potential biomarkers, associating gene modules with clinical features, conducting functional enrichment analysis, and constructing gene regulatory networks. In this study, we specifically selected the intersection genes of T cell-related genes and ubiquitination-related genes to construct a gene co-expression network. Subsequently, we screened for modules closely associated with survival time for further analysis.

### 2.4 Modeling construction and validation

We utilized the lasso algorithm to select prognosis-related key genes and employed Cox analysis to construct a prognostic model (20). Through this model, we calculated the riskscore for each patient, followed by a comprehensive assessment of the roles of these key molecules in the prognosis of liver cancer patients.

The riskscore for each liver cancer patient was calculated using the following formula:


$$\begin{array}{c} \text{riskscore}=\left[\text{Expression of UBE}2\text{E}1\times \text{coefficient}\right]\;+\;\left[\text{Expression of PSMD}1\times \text{coefficient}\right]+\\ \left[\text{Expression of FBXL}5\times \text{coefficient}\right]\\ +\left[\text{Expression of RNF}10\times \text{coefficient}\right]\\ +\\ \left[\text{Expression of IVNS}1\text{ABP}\times \text{coefficient}\right] \end{array}$$

The TCGA dataset was partitioned into training and validation subsets, with additional validation performed using the GSE14520 dataset. Kaplan-Meier survival curve analysis was conducted to assess the prognosis of patients in different groups. The risk survival curve was utilized to evaluate the survival and death statuses of patients in the high and low-risk groups. We also employed heatmaps to illustrate the differences in key genes between the two groups in the constructed model. ROC curves were employed to determine the predictive performance of the model’s riskscore and various clinical features for disease prediction. Cox regression was utilized to evaluate the predictive performance of the model’s riskscore and various clinical features for disease prognosis. The nomogram was constructed using riskscore and various clinical features to assess the prognosis of different patients at 1, 3, and 5 years. Calibration curves were used to predict whether the predictive performance of the clinical model was close to the actual situation. ROC curves and KM curves further validated the reliability of the model.

### 2.5 Immunoassay

This study employed the ssGSEA algorithm to accurately calculate the infiltration abundance of 28 immune cell types in each liver cancer patient and analyzed the correlation between riskscore and immune cells (21, 22). MSI, as an important prognostic factor and treatment target in tumors, its score differences between high and low-risk groups can effectively predict the efficacy of immunotherapy. Meanwhile, we introduced the tumor stemness index as an indicator to evaluate the similarity between tumor cells and stem cells, and predicted the degree of tumor dedifferentiation based on the differences in stemness scores between high and low-risk groups. To further evaluate the relationship between riskscore and immunotherapy, we conducted Spearman correlation analysis, calculated the correlation between riskscore and MMR and immune checkpoints, thus evaluating the potential value of riskscore in predicting the efficacy of immunotherapy (20). In addition, we also utilized the IMgivor210 dataset, which records the sequencing data and clinical information of patients receiving PD-L1 inhibitor treatment, to predict the efficacy of immunotherapy in different liver cancer patients.

### 2.6 Drug sensitivity analysis and mutation analysis

Chemotherapy is a treatment method that uses chemical drugs to treat cancer and other diseases. Chemotherapeutic drugs can intervene in the growth and division of cancer cells in different ways, thereby inhibiting the proliferation or killing of cancer cells. We used the “oncopredict” package to evaluate the sensitivity of 8 chemotherapy drugs related to liver cancer, including 5-Fluorouracil, Camptothecin, Cisplatin, Sorafenib, Vinblastine, Oxaliplatin, Gemcitabine, Irinotecan (23, 24). The effectiveness of drugs was predicted by analyzing the IC50 scores between high and low-risk groups. Mutation analysis is the process of detecting, describing, classifying, and interpreting mutations that occur in biological samples. In mutation analysis, SNP (Single Nucleotide Polymorphism), ONP (Single Nucleotide Variation), Ins (Insertion), and Del (Deletion) are common types of mutations, representing different types of DNA changes. Analyzing the types of mutated genes in high and low-risk group patients can help understand the molecular mechanisms of liver cancer.

### 2.7 Real-time quantitative PCR

Detailed steps can be found in previous research (25). In short, total RNA from cells was obtained using TRIZOL, then cDNA synthesis was conducted using PrimeScript™ RT Master Mix (Takara Bio, Japan) followed by RT-PCR utilizing TB Green (Takara, Japan), with GAPDH serving as the internal reference gene. Detailed primer sequences employed in this investigation are listed in **Supplementary Table 1**.

### 2.8 Cell culture and cell transfection

The hepatocellular carcinoma cell lines HCCLM3 and BEL7402 were obtained from the Cell Bank of the Chinese Academy of Sciences (Shanghai, China). Cell transfection was performed according to the manufacturer’s instructions of jetPRIME transfection reagent (Polyplus, China). The siRNA sequences used in this study are as follows: siUBE2E1–1: sense-GCCUCCAAAGGUUACAUUU; antisense-AAAUGUAACCUUUGGAGGC. siUBE2E1–2: sense-CAAAGGCGAUAACAUCUAU; antisense-AUAGAUGUUAUCGCCUUUG.

### 2.9 CCK8 assay

1500 HCC cells were seeded in a 96-well plate with 5 replicate wells (Corning, USA). At 0, 24, 48, 72, and 96 hours of cell culture, 10 μL of CCK8 working solution was added (Targetmol, USA), and after 2 hours of incubation in the cell culture incubator, absorbance was measured at 450 nm wavelength using a microplate reader.

### 2.10 Transwell assay

A total of 50,000 HCC cells were inoculated into the upper chamber of a Transwell insert (Corning, USA), with serum-free medium in the chamber and 750 ml of complete medium in the lower chamber. Matrigel was added or not added to the bottom of the upper chamber for migration and invasion experiments. After 24 hours of incubation, the experiment was terminated. Following fixation with 4% paraformaldehyde for 15 minutes, the cells were subsequently subjected to staining with crystal violet. Carefully wipe the cells on the upper chamber with a cotton swab, and photograph and record under a microscope, the magnification used was 100X. Subsequently, cell counting analysis was performed using ImageJ software.

### 2.11 Data statistics

Differential analysis between groups was conducted using the Wilcoxon test, while correlation analysis was performed using the Spearman correlation test. Survival analysis between groups was carried out using Kaplan-Meier analysis and the log-rank test. Cox regression analysis was conducted using the “survival” package in R software to calculate hazard ratios (HRs) and their 95% confidence intervals (CIs). All statistical tests were two-sided, with statistical significance set at P < 0.05. The analyses were performed using R software (version 4.2.2).

## 3 Results

### 3.1 Single-cell sequencing analysis

**Figure 1:**
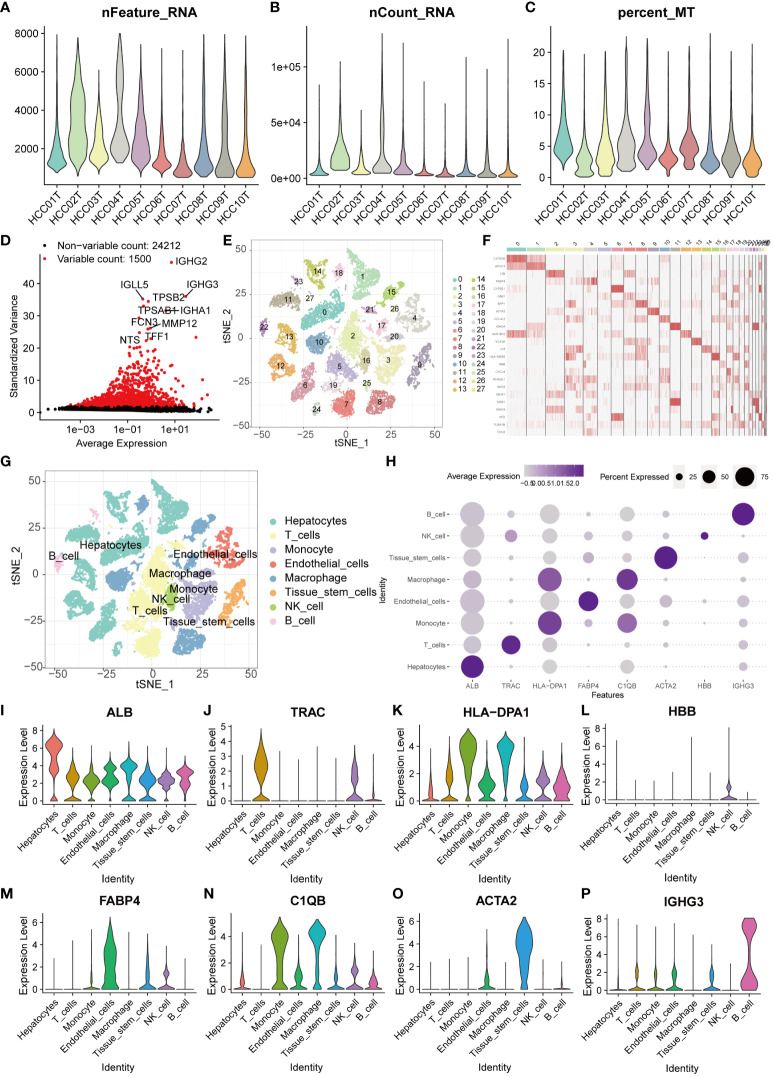
Single-cell sequencing analysis for screening T cell-related genes. **(A–C)** The violin plots show the nFeature_RNA, nCount_RNA, and percent_MT of 10 HCC samples. **(D)** The volcano plot displays 1500 highly variable genes. **(E)** PCA and tSNE clustering divided cells into 28 clusters. **(F)** The heatmap displays the major differentially expressed genes in different clusters. **(G)** The singleR package annotated cells and categorized them into 8 major cell groups. **(H)** The bubble plot illustrates the major differentially expressed genes in different cell types. **(I–P)** The violin plots show the major differentially expressed genes in different cell types.

We obtained single-cell sequencing data (GSE149614) of liver cancer patients from the GEO database. After rigorous screening and organizing, a total of 10 liver cancer samples were selected for subsequent in-depth analysis. Initially, we extensively discussed the nFeature and nCount of each sample and calculated the percentage of mitochondrial gene expression in each cell relative to the total gene expression (**Figures 1A–C**). Based on these indicators, we established screening criteria (nFeature_RNA > 50 & percent_MT < 5%) to filter out cells of good quality, ensuring the accuracy of subsequent analysis. Furthermore, we extracted genes with a large coefficient of variation between cells, totaling 1500 major highly variable genes (**Figure 1D**). These genes exhibit significant expression differences among single cells, thus being considered critical factors influencing cell type discrimination. Using these genes, we conducted principal component analysis and tSNE dimensionality reduction. Through clustering analysis, the samples were successfully divided into 28 different cell clusters (**Figures 1E, F**). Subsequently, we conducted comprehensive annotation work on these cells. The results showed that the cells were accurately classified into eight major categories, including hepatocytes, T cells, monocytes, endothelial cells, macrophages, tissue stem cells, natural killer cells, and B cells (**Figure 1G**). To visually demonstrate the major differential genes between different cell types, we generated a bubble plot for visualization (**Figure 1H**). The major differential genes in Hepatocytes are ALB, in T cells are TRAC, in Monocytes are HLA-DPA1, in Endothelial cells are FABP4, in Macrophages are C1QB, in Tissue stem cells are ACTA2, in NK cells are HBB, and in B cells are IGHG3 (**Figures 1I–P**).

### 3.2 Construction and efficacy validation of model using WGCNA combined with LASSO-COX algorithm

**Figure 2:**
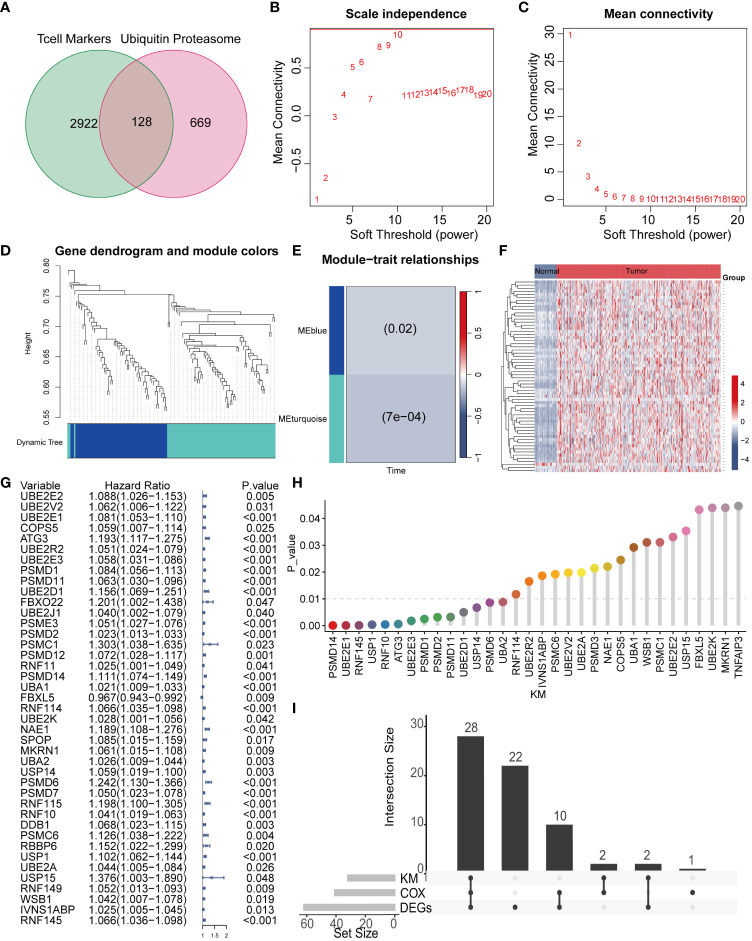
WGCNA combined with differential and prognosis analysis to identify key genes. **(A)** The Venn diagram illustrates the intersection of T cell marker genes and ubiquitin proteasome system genes. **(B, C)** The WGCNA algorithm demonstrates the optimal soft threshold. **(D)** The gene dendrogram displays genes are well clustered into 2 categories. **(E)** MEturquoise module genes are found to be closely associated with survival time. **(F)** The heatmap shows the differential genes of the MEturquoise module between cancer tissues and normal tissues. **(G)** COX analysis shows 41 genes with prognostic value. **(H)** KM analysis shows 32 genes with prognostic value. **(I)** The UpSet plot shows the intersection of differential analysis, KM analysis, and COX analysis with 28 genes.

Given the pivotal role of T cells in the occurrence and development of liver cancer, we screened 3050 genes closely associated with T cells (26). Ubiquitination, as a vital biochemical process post protein synthesis, profoundly influences biological processes such as cell growth, differentiation, cell cycle regulation, apoptosis, and cancer occurrence by chemically modifying amino acid residues. We extracted a total of 797 key genes closely related to ubiquitination modification. By comparing the two sets of key genes, we identified 128 common genes, which we defined as TCRUG. These genes play important roles in both T cell function and ubiquitination modification processes (**Figure 2A**). To delve deeper into the functional network of TCRUG in liver cancer, we performed WGCNA analysis. The results revealed a clear division of samples into two modules (**Figures 2B–D**). Subsequently, we specifically examined a module highly correlated with survival time-the MEturquoise module, where gene expression may be tightly linked to the prognosis of liver cancer patients (**Figure 2E**). Afterwards, we performed differential expression analysis (P<0.05), Kaplan-Meier survival analysis (P<0.05), and Cox regression analysis (P<0.05) on the genes within this module to unveil their potential mechanisms in the occurrence and development of liver cancer (**Figures 2F–H**). Finally, through the integration of the aforementioned analysis results, we identified 28 common genes, which will serve as the focal points for subsequent research (**Figure 2I**).

**Figure 3:**
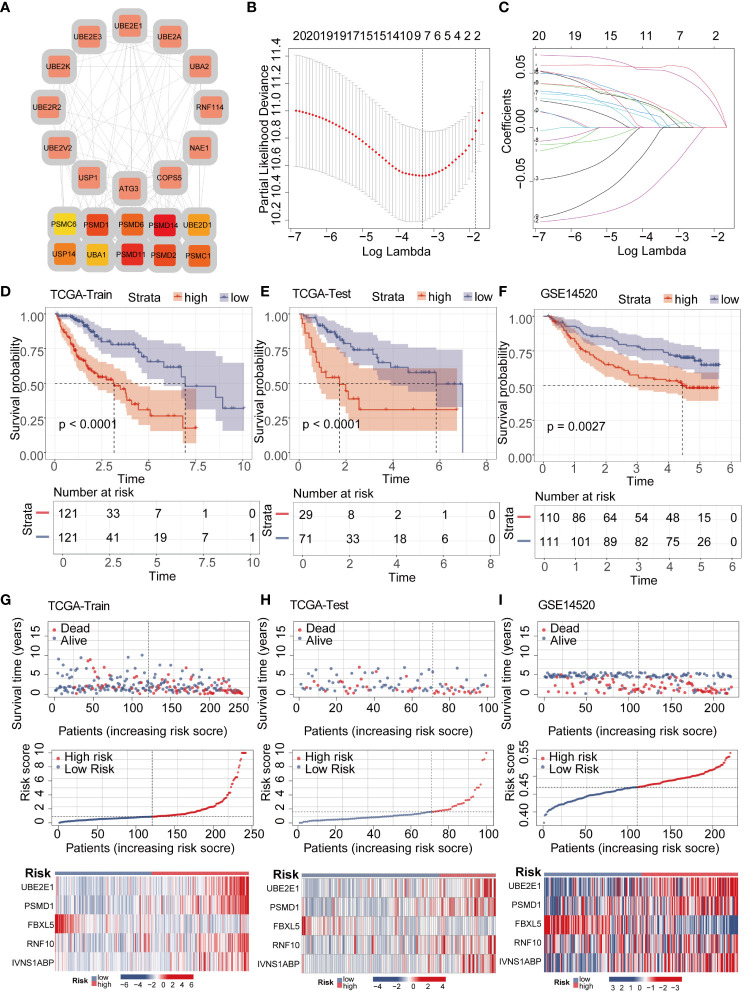
LASSO-COX algorithm constructs a risk prognosis model and validation. **(A)** The PPI network shows the correlation and importance of key genes. **(B, C)** Genes suitable for constructing the optimal model were selected using the LASSO-COX algorithm. **(D–F)** KM analysis revealed that patients in the high-risk group had a worse prognosis than those in the low-risk group in different datasets. **(G–I)** Survival analysis revealed a higher mortality rate in the high-risk group, and the heatmap demonstrated higher expression levels of UBE2E1, PSMD1, RNF10, and IVNS1ABP in the high-risk group, while FBXL5 exhibited higher expression levels in the low-risk group.

**Figure 4:**
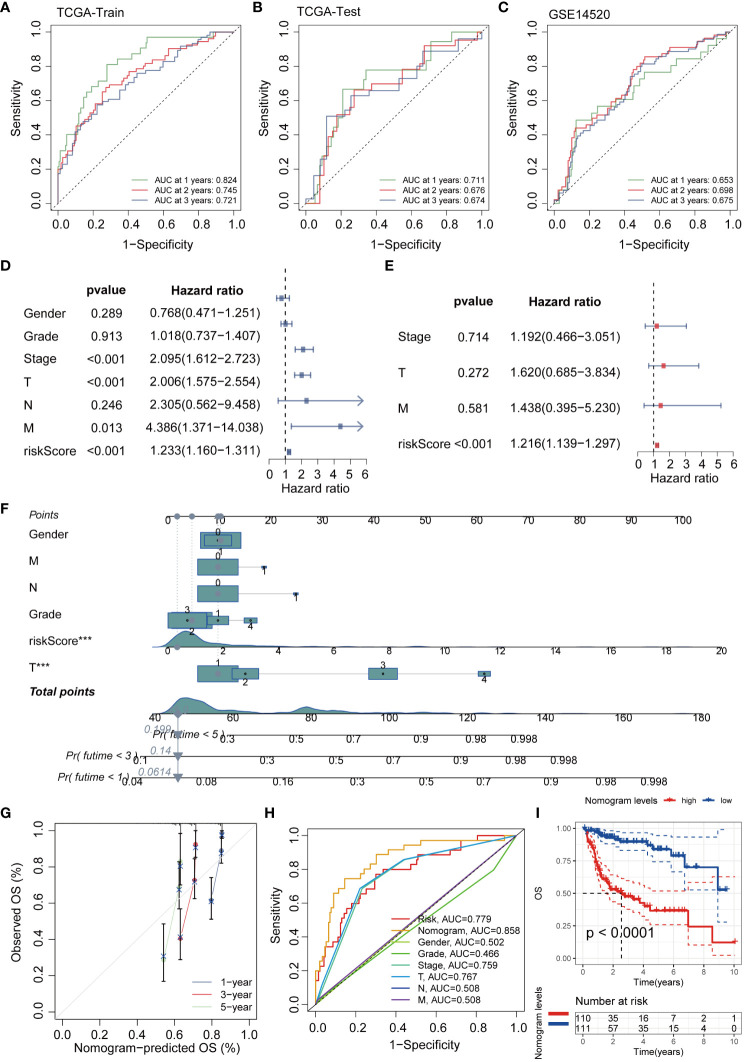
The efficacy validation of the riskscore model and the construction and validation of clinical predictive models. **(A–C)** ROC curves show the AUC values for patients in different datasets at 1, 3, and 5 years. **(D, E)** Univariate and multivariate COX analyses revealed that the riskscore is a valuable independent prognostic factor. **(F)** The nomogram was constructed by integrating the riskscore and clinical factors to predict patient survival at 1, 3, and 5 years. **(G)** The calibration curve illustrates that the model can reasonably predict patient survival. **(H)** ROC curves demonstrate that the AUC value of the nomogram score can reach 0.858. **(I)** KM analysis revealed that patients with high nomogram scores had a worse prognosis.

To further explore the interaction relationships among the intersection genes, we conducted detailed analysis using the STRING database (**Figure 3A**). Subsequently, by applying the lasso-cox algorithm, we accurately screened out five key genes-UBE2E1, PSMD1, FBXL5, RNF10, and IVNS1ABP, which were used to construct the prognostic model (**Figures 3B, C**). Due to the tight association between riskscore and patient prognosis, we employed three different datasets to thoroughly validate the prognostic predictive efficacy of the model. Utilizing the R package “caret,” we partitioned the TCGA dataset into training and validation sets, while introducing the GSE14520 dataset as an additional validation set. Analysis results demonstrated that patients with high riskscore exhibited poorer prognosis trends across these three datasets (**Figures 3D–F**). To further explore survival outcomes across different risk groups, comprehensive comparative analyses were conducted. The findings indicated a significantly higher mortality rate among patients in the high-risk group compared to those in the low-risk group. Additionally, heatmap analysis revealed distinct expression patterns among the genes utilized for modeling, including UBE2E1, PSMD1, RNF10, and IVNS1ABP, which exhibited markedly elevated expression levels in the high-risk group compared to the low-risk group. Conversely, FBXL5 displayed lower expression levels in the high-risk group relative to the low-risk group (**Figures 3G–I**). To assess the performance of the classification model, we employed ROC curves for analysis. The AUC value, representing the area under the ROC curve, served as a crucial metric to gauge the performance of the classifier. In the TCGA training set, the AUC values reached 0.824, 0.745, and 0.721, respectively; whereas in the TCGA validation set, the AUC values were 0.711, 0.676, and 0.674, respectively. Moreover, the AUC values of the GSE14520 validation set also exhibited good performance, with values of 0.653, 0.698, and 0.675, respectively (**Figures 4A–C**). To delve deeper into the relationship between riskscore and clinical characteristics, we conducted univariate and multivariate COX prognostic analyses. Univariate COX analysis revealed that Stage, T, M stage, and riskscore all held significant prognostic value. Moreover, multivariate COX regression analysis provided further evidence that riskscore served as a valuable independent prognostic factor (**Figures 4D, E**). Taking into account the potential improvement in prognostic accuracy through the amalgamation of clinical parameters with riskscore, we developed a nomogram to predict the prognosis of patients at 1, 3, and 5 years (**Figure 4F**). Calibration curve validation illustrated the robust predictive performance of this index. Further ROC curve analysis revealed an AUC value as high as 0.858. Additionally, KM analysis suggested that patients with higher Nomogram scores exhibited poorer prognosis (**Figures 4G–I**).

### 3.3 Immune landscape of the riskscore model

**Figure 5:**
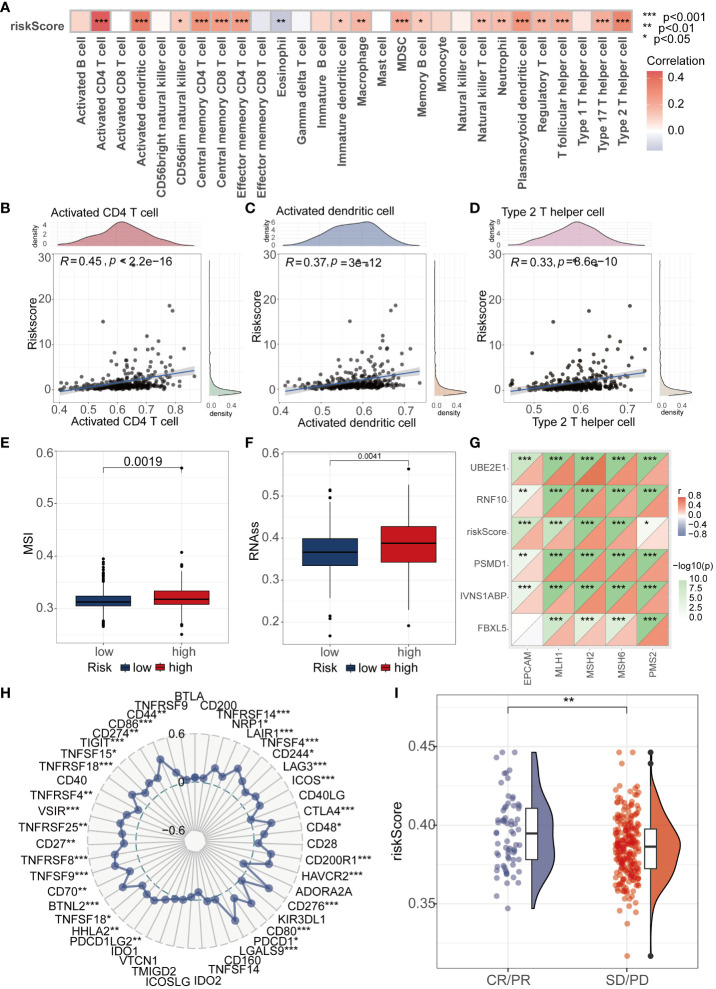
Analysis of Riskscore and Immune Landscape. **(A)** The correlation between riskscore and 28 immune cells was calculated using the ssGSEA algorithm. **(B–D)** Correlation analysis found that Activated CD4 T cell, Activated dendritic T cell, and Type 2 helper cell had the highest correlation with riskscore (P<0.001, R>0.3). **(E)** Differential analysis found that the MSI score was higher in the high-risk group. **(F)** Differential analysis found that the high-risk group had a higher tumor stemness score. **(G)** MMR genes were found to be closely associated with riskscore. **(H)** Radar plots showed the correlation between riskscore and multiple immune checkpoints. **(I)** Patients with higher riskscore were more likely to experience remission according to the IMvigor210 dataset. * represents p < 0.05, ** represents p < 0.01, and *** represents p < 0.001.

In order to further investigate the potential association between riskscore and immune cells, we conducted comprehensive analysis of 28 immune cells using the ssGSEA algorithm. The results revealed significant positive correlations between riskscore and Activated CD4 T cell, Activated dendritic T cell, and Type 2 helper cell (P<0.001, R>0.3) (**Figures 5A–D**). Furthermore, an evaluation of the MSI score revealed elevated scores in the high-risk group compared to the low-risk group (**Figure 5E**). Tumor stemness serves as a crucial parameter for gauging the resemblance of tumor cells to stem cells. Our findings indicated a markedly elevated tumor stemness score among patients in the high-risk group compared to those in the low-risk group (**Figure 5F**). To further investigate the differences between high and low-risk groups in terms of immunotherapy, we utilized the analysis of MMR (mismatch repair) related genes and immune checkpoint markers to predict the correlation between riskscore and immunotherapy. During the MMR analysis, a notable positive correlation was identified between the riskscore and the expression levels of pivotal genes, including EPCAM, MLH1, MSH2, MSH6, and PMS2 (**Figure 5G**). We further analyzed the immune checkpoint-related markers. The results revealed a close association between immune checkpoint molecules such as CTLA4, PD1, and PDL1 and riskscore (**Figure 5H**). To provide a more intuitive demonstration of the differences in immunotherapy between high and low-risk groups, we conducted an analysis of immunotherapy scores. The results showed that patients in the high-risk group exhibited a more significant effect in immunotherapy (**Figure 5I**).

### 3.4 Mutation analysis and drug sensitivity analysis of the riskscore model

**Figure 6:**
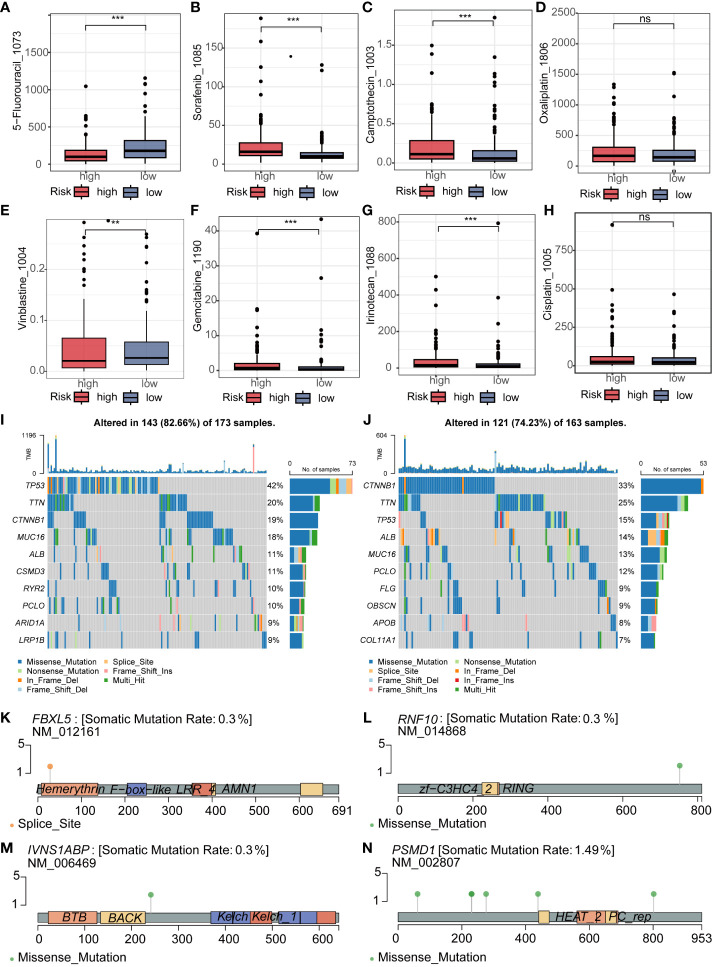
Analysis of Drug Sensitivity and Mutation in Riskscore. **(A–H)** The IC50 of 5_Fluorouracil and Vinblastine was lower in the low-risk group, while it was lower for Sorafenib, Camptothecin, Gemcitabine, and Irinotecan in the high-risk group. There was no significant difference in the IC50 between the two groups for Oxaliplatin and Cisplatin. **(I, J)** The waterfall plot revealed different mutated genes and mutation rates between the high and low-risk groups. **(K–N)** Mutation rates and mutation analysis of the four key genes in the model. ns represents p > 0.05, ** represents p < 0.01, and *** represents p < 0.001.

Subsequently, we delved into the differences in sensitivity to eight different hepatocellular carcinoma (HCC) chemotherapeutic drugs between the high and low-risk groups. It is noteworthy that we found samples in the high-risk group exhibited significantly lower IC50 values for 5_Fluorouracil and Vinblastine compared to the low-risk group, indicating higher sensitivity. In contrast, Sorafenib, Camptothecin, Gemcitabine, and Irinotecan showed significantly higher IC50 values in the high-risk group compared to the low-risk group. However, there were no significant differences in sensitivity between the high and low-risk groups for Oxaliplatin and Cisplatin (**Figures 6A–H**). To comprehensively investigate the variations in gene mutations between high and low-risk groups, we conducted detailed analyses utilizing mutation data retrieved from the TCGA database. Additionally, to visually illustrate these differences, we generated waterfall plots and applied a threshold mutation rate greater than 10% for filtering purposes. The analysis outcomes indicated notably elevated gene mutation rates for TP53, MUC16, CSMD3, and RYR2 in the high-risk group compared to the low-risk group. Conversely, CTNNB1, TTN, ALB, and PCLO exhibited higher gene mutation rates in the low-risk group (**Figures 6I, J**). To obtain a more refined insight into the mutation status of pivotal genes within the established model, we performed separate analyses of somatic mutation rates for each gene. The results indicated that the mutation rates of FBXL5, RNF10, and IVNS1ABP were all 0.3%, whereas PSMD1 had a mutation rate of 1.49% (**Figures 6K–N**).

### 3.5 The oncogenic role of UBE2E1 in HCC

**Figure 7:**
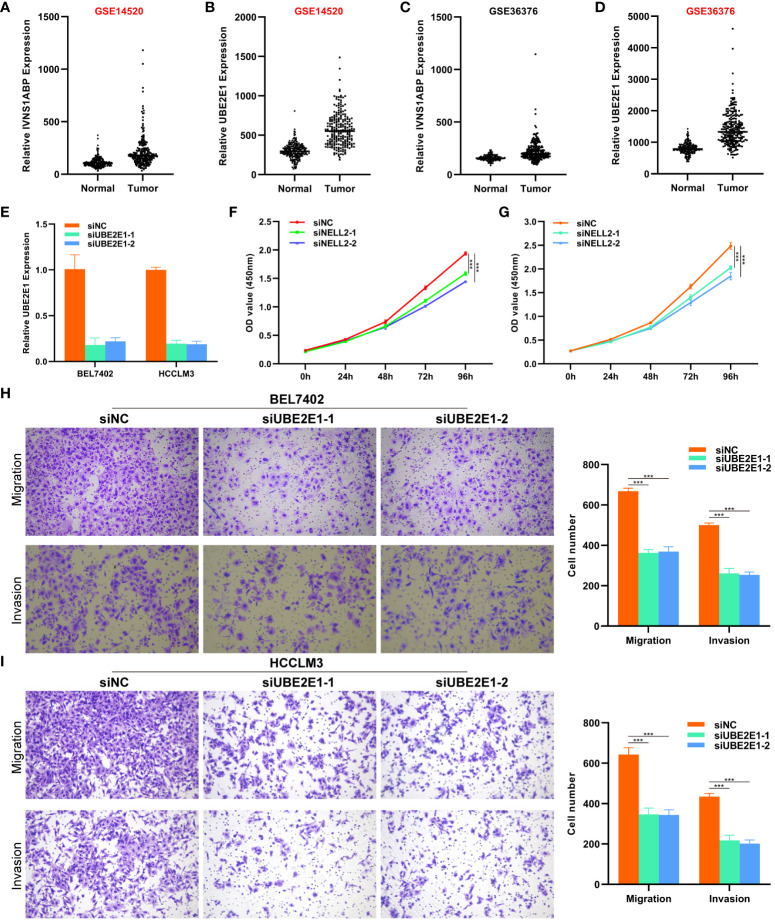
CCK8 and Transwell experiments revealed that UBE2E1 promotes proliferation and migration of HCC. **(A–D)** UBE2E1 was found to be expressed higher in tumor tissues than in normal tissues in both datasets. **(E)** RT-qPCR validation confirmed that UBE2E1 was stably knocked down in BEL7402 and HCCLM3 cell lines. **(F, G)** CCK8 experiments revealed that knocking down UBE2E1 could inhibit the proliferation of liver cancer cells. **(H, I)** Transwell experiments revealed that knocking down UBE2E1 could inhibit the migration ability of liver cancer cells. *** represents p < 0.001.

Considering the research gap of UBE2E1 and IVNS1ABP in HCC, we conducted further studies on these two genes. We investigated the expression differences of UBE2E1 and IVNS1ABP in HCC in two external datasets (GSE14520 and GSE36376). We found that UBE2E1 was significantly differentially expressed in both datasets (Log2Foldchange >0.585 and P < 0.05) (**Figures 7A–D**). Hence, UBE2E1 was ultimately chosen as the object of our in-depth investigation. First, we constructed cell lines with UBE2E1 knockdown in BEL7402 and HCCLM3 cell lines (**Figure 7E**). CCK8 and colony formation experiments indicated that the proliferation ability of HCC cells was significantly impaired after UBE2E1 knockdown (**Figures 7F, G**). Transwell assays demonstrated that the migration and invasion abilities of HCC cells were significantly impaired after UBE2E1 knockdown (**Figures 7H, I**).

## 4 Discussion

HCC is often diagnosed at an advanced stage due to its insidious onset, and treatment options for advanced HCC patients are limited and ineffective (27)。Moreover, HCC is a highly heterogeneous disease, and due to the significant differences in the TME, the treatment outcomes may vary greatly even with the same therapeutic approach (28)。Therefore, it is necessary to deeply analyze the TME of HCC, especially the immune cells within the TME (29). In this study, we analyzed cell subtypes at the single-cell level using scRNA-seq data from HCC patients. scRNA-seq data from 10 HCC patients were downloaded from the GEO database (GSE149614), and after analyzing cell subtypes, the focus of the study was placed on T cells closely associated with tumor progression. Developing biomarkers based on T cells to accurately predict the prognosis and immunotherapy response of HCC patients is highly promising and important.

In this study, we obtained 3050 T cell marker genes. In addition, we incorporated 797 ubiquitination family molecules from Bulk RNA data of HCC patients, finally identifying 128 TCRUG In order to explore the function and prognostic value of these TCRUG in depth, we conducted a series of comprehensive analyses, including differential analysis, weighted gene co-expression network analysis (WGCNA), and prognostic analysis. Ultimately, we identified 5 core T cell marker genes, including UBE2E1, PSMD1, FBXL5, IVNS1ABP, and RNF10. Among them, PSMD1 can affect HCC cell proliferation and apoptosis by influencing lipid droplet formation (30). FBXL5 can prevent iron overload and inhibit HCC occurrence on the one hand (31), and inhibit HCC metastasis by suppressing snail expression levels on the other hand (32). In another study, RNF10 has been confirmed to be a core gene predicting the prognosis of HCC patients, while UBE2E1 and IVNS1ABP have no studies in HCC. Considering the research gap of UBE2E1 and IVNS1ABP in HCC and the stability of UBE2E1 expression difference in HCC, we focus on UBE2E1 and demonstrate its carcinogenic effect in HCC through a series of *in vitro* cell experiments.

Around these 5 core TCRUG, we constructed a novel riskscore. The riskscore demonstrated stable and accurate prediction of HCC patient prognosis in the development cohort, internal validation cohort, and external validation cohort, namely, patients in the high-risk group had poorer overall survival. In addition, based on the riskscore and common clinical parameters, we constructed a column diagram that is easy to use in clinical practice. We also utilized calibration curves and ROC evaluation to assess the stability and accuracy of the column diagram in predicting HCC overall survival.

To understand the correlation between riskscore and tumor immunotherapy response, we conducted multidimensional exploration. we investigated the association between the riskscore and immune cell infiltration levels. Our findings revealed a positive correlation between the riskscore and the majority of immune cell types. Particularly noteworthy was the robust correlation observed with Activated CD4 T cells, Activated dendritic T cells, and Type 2 helper cells. These results suggest that high-risk patients with HCC exhibit higher immune cell infiltration in tumor tissues, indicating a propensity for hot tumor classification (33). Additionally, we found that higher riskscore were associated with higher levels of MSI. This result sparked our interest. Previous studies have shown that higher MSI levels may lead to better immunotherapy outcomes (34). Therefore, we hypothesize that patients in the high-risk group may have better immunotherapy efficacy due to higher MSI, despite their poorer prognosis. Furthermore, we found that patients in the high-risk group had higher tumor stemness. MMR(Mismatch Repair)MMR (Mismatch Repair), which corrects base-pairing errors during DNA replication, is closely related to the efficacy of immunotherapy (35, 36). Correlation analysis revealed a close association between riskscore and multiple MMR genes, indicating that the riskscore is a good predictor of immunotherapy response. The core of immune checkpoint therapy is to use specific inhibitors to suppress the function of immune checkpoints, thereby enhancing immune response and eliminating tumor cells (37, 38). Therefore, analyzing the correlation with immune checkpoints can predict the efficacy of immunotherapy. We found that the riskscore was positively correlated with 32 immune checkpoint molecules (out of 48 in total), suggesting that the riskscore is a promising new indicator of immune response. In the IMgivor210 cohort, we observed that the riskscore was elevated in the immune response group (CR/PR) compared to the non-immune response group (SD/PD), aligning with our earlier findings from the MSI analysis. Taken together, these consistent observations suggest that individuals classified into the high-risk group may exhibit enhanced immune responses following immunotherapy.

Repurposing old drugs for cancer treatment provides a new perspective. On one hand, it saves a lot of time and economic costs in developing new drugs. Furthermore, adverse drug reactions have been thoroughly studied. In this study, we demonstrated differences in sensitivity to 8 drugs among different risk groups. These include classic drugs such as sorafenib and 5-fluorouracil. These results provide new insights for personalized treatment of HCC patients.

Of course, our study has several limitations that need to be acknowledged. First, bioinformatics relies on high-quality data. Low-quality experimental data, sequencing errors, etc., can affect the accuracy and reliability of the analysis. Second, we have not further explored the downstream mechanisms of the modeling genes, which may affect the accuracy of our predictions of immunotherapy efficacy and targeted drugs. Therefore, further in-depth research is needed.

In conclusion, this study developed a novel riskscore based on the close interaction between T cells and ubiquitination modification. This riskscore can accurately and stably predict the prognosis and immunotherapy response of HCC patients. Additionally, our findings suggest that inhibiting UBE2E1 expression can suppress the proliferation, migration, and invasion ability of HCC cells.

## Data availability statement

The datasets presented in this study can be found in online repositories. The names of the repository/repositories and accession number(s) can be found in the article/**Supplementary Material**.

## Ethics statement

Ethical approval was not required for the studies on humans in accordance with the local legislation and institutional requirements because only commercially available established cell lines were used. Ethical approval was not required for the studies on animals in accordance with the local legislation and institutional requirements because only commercially available established cell lines were used.

## Author contributions

CC: Data curation, Formal analysis, Investigation, Software, Writing – original draft. ZC: Conceptualization, Formal analysis, Investigation, Resources, Writing – original draft. ZZ: Methodology, Supervision, Validation, Writing – original draft. HY: Formal analysis, Project administration, Validation, Writing – original draft. SX: Formal analysis, Validation, Writing – original draft. WH: Resources, Visualization, Writing – original draft. ZX: Project administration, Resources, Writing – original draft. CG: Methodology, Project administration, Writing – original draft. CZ: Validation, Visualization, Writing – original draft. DY: Data curation, Project administration, Writing – original draft, Writing – review & editing. JS: Investigation, Project administration, Software, Validation, Writing – original draft, Writing – review & editing.
